# Data on the current state of problem solving and improvement during physical product development within complex (manufacturing) systems

**DOI:** 10.1016/j.dib.2019.103851

**Published:** 2019-03-20

**Authors:** Peter Burggräf, Tim Weißer, Johannes Wagner

**Affiliations:** University of Siegen, Siegen, Germany

**Keywords:** Manufacturing problem solving, Product development, Knowledge based systems

## Abstract

Due to the exponential increase of failure cost during the product development process, problems have to be effectively remedied as early as possible and with shortened innovation cycles, increasingly efficient. For the manufacturing of complex products at low maturity levels (referred to as physical product development), nonconformance problem solving constitutes a major difficulty in this regard (Camarillo et al., 2017; Walter et al., 2010). The data presented in this article was collected from German companies, differing in size and industry sector, manufacturing highly complex products at low maturity. Selected and consulted companies therefore operate in (or comparable to) the automotive prototyping, air and space, shipbuilding, special machinery or electronics domain. The survey comprises the answers of 46 participants, gathered via online questionnaire. It subdivides into 18 questions covering the companies’ characteristics, knowledge management and documentation systems within the product development process, as well as the appraisal of technological potentials. The obtained data gives an insight into the industrial status quo of nonconformance problem solving. The data allows to derive existing deficits and dedicated research on solutions.

**Table:** Specifications table

Subject area	*Industrial and Manufacturing Engineering, Knowledge Management.*
More specific subject area	*Manufacturing Problem Solving, Nonconformance Problem Solving, Quality Management.*
Type of data	*Tables, figures*
How data was acquired	*Consultation of relevant participants and survey* via *online questionnaire. Questions and prescribed answer options are part of the attached data.*
Data format	*Raw, analyzed*
Experimental factors	*Survey data of 46 respondents covering 18 questions on problem related knowledge management and documentation systems.*
Experimental features	*Companies manufacturing complex products at low maturity levels. Respondents in charge of product development, production or product quality (*cf. *Automotive prototyping, air and space, shipbuilding, special machinery or electronics domain).*
Data source location	*Germany*
Data accessibility	*A comprehensive dataset is attached to this data article as Microsoft Excel spreadsheet.*
Related research article	*A. Camarillo, J. Ríos, K.D. Althoff, CBR and PLM applied to diagnosis and technical support during problem solving in the Continuous Improvement Process of manufacturing plants, Procedia Manufacturing, 13, 2017, 987–994*[1].

**Table:** 

**Value of the data** • While lean product development and ramp-up as upstream respectively downstream phases to the field of observation, already gained a lot of attention within the research community, the presented data allows an insight into the rather uninvestigated field of problem solving within physical product development. • The data characterizes problem relevant knowledge acquisition, process wide accessibility versus usage and puts it in context with the ability to remedy problems efficiently and effectively. • The data describes the current status quo of problem solving within physical product development in practice. Used as an explanation component, the data allows to reveal deficits and opportunities which may be addressed by dedicated research.

## 1 Data

The survey was conducted from July until September 2018 via online questionnaire, comprising 18 questions and was answered by *n =* 46 participants of companies in the German industry sector. The questionnaire is divided into a first section to collect demographic information and a second section, collecting data on problem solving in the physical product development domain. The data predominantly consists of ordinally scaled variables, either representing the participants’ degree of consent to a statement, or a self-assessment of their situation and capabilities. The full Dataset (Q1-18) is provided as supplementary material in [dataset] Appendix A.

## 2 Experimental design, materials, and methods

**Fig. 1:**
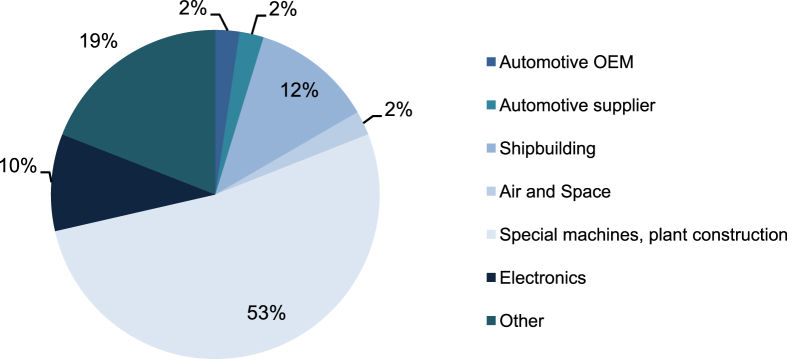
Segmentation of the surveyed companies by industry sector.

To capture the status quo of problem solving and improvement during physical product development within complex (manufacturing) systems, the survey's target group was intended to manufacture complex products at low maturity. This applies to small lot sizes (down to one) produced to order, as well as prototype series, hedging large-scale production. Selected and consulted companies with high product and process complexity operate in (or comparable to) the automotive prototyping, air and space, shipbuilding or special machinery or electronics domain. Nonconformance problem solving in such domains constitutes a major part of operations and requires the integration of distributed knowledge [2]. Respondents were supposed to be in charge of product development, production/quality control or technical sales. A segmentation of the participating companies is given in Fig. 1. Half of the companies classify themselves as Small or Medium Enterprise (SME, < 500 employees and <50 million € sales [3]), 24% are below 500 million € in sales and 26% above that. On weighted average, the questioned companies assessed the complexity of their products and processes as high.

**Fig. 2:**
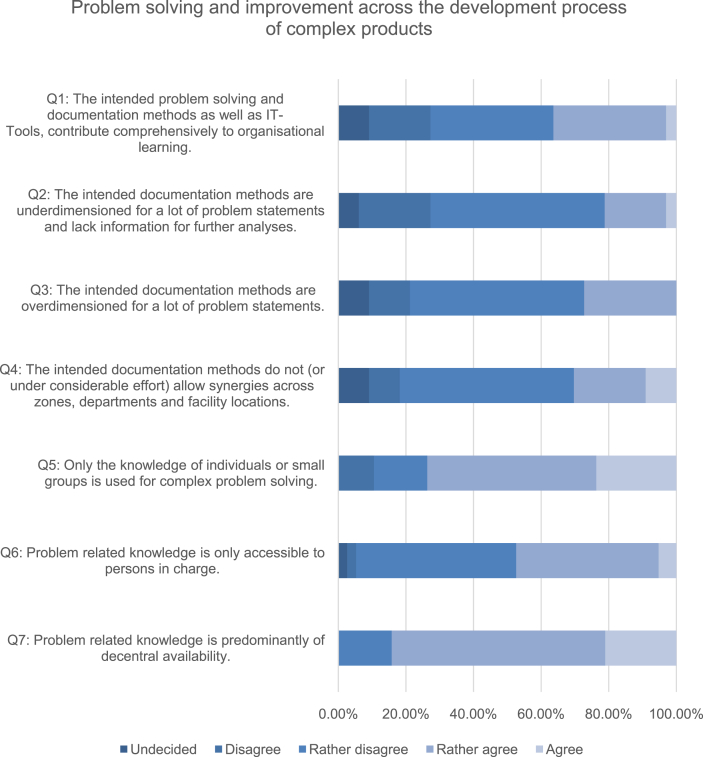
Overview evaluation for the questions answered by degree of consent (statements shortened).

More than 50% of the respondents judge their problem solving efforts caused by documentation, communication and the search for responsibility as rather high, 13% even as very high. An overview evaluation for questions on problem documentation methods as well as the availability and usage of problem related knowledge is presented in Fig. 2 (For clarity reasons the subsequent numbering differs from the original numbering within the questionnaire). Although documentation systems are well dimensioned and would potentially allow intra-organizational synergies, which the major part of the respondents declared (cf. Q1-4), problem related knowledge is of predominantly decentral availability (cf. Q7). Complex problems are solved based on the knowledge of individuals or small groups, instead of organizational knowledge, as 74% of the participants agreed.

**Fig. 3:**
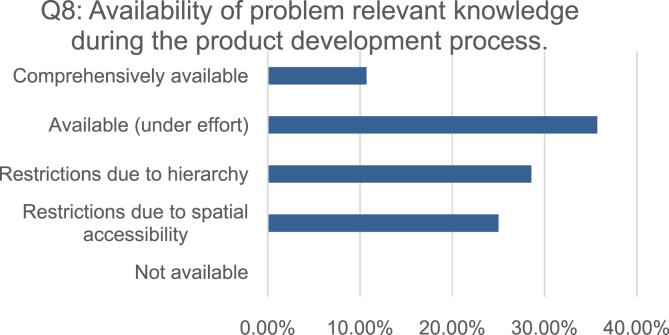
Distribution of the self-assessments regarding the availability of problem relevant knowledge across the product development process.

**Fig. 4:**
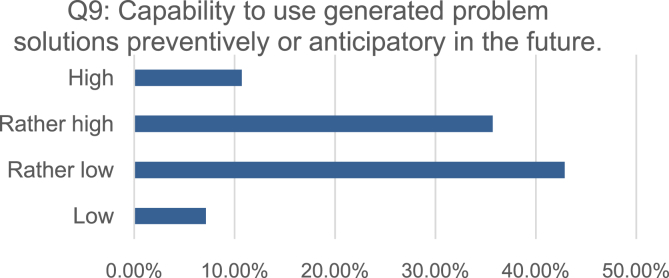
Distribution of the self-assessments regarding the capability to use historical problem solutions to develop prevention or anticipation mechanisms.

**Fig. 5:**
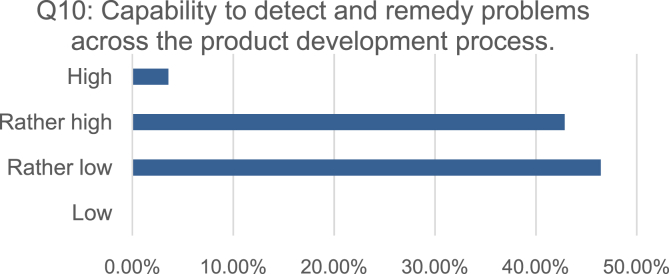
Distribution of the self-assessments regarding the overall capability to eliminate problems during the product development process.

To analyze the effects of knowledge availability (Q8, Fig. 3), documentation methods (Q4, Fig. 2), knowledge utilization (Q5, Fig. 2) and lessons learned (Q9, Fig. 4) upon the capability to holistically detect and remedy problems across the product development process (Q10, Fig. 5), the ordinally scaled variables were tested via Spearman rank correlation.

The calculations of the rank correlation for tied ranks are presented in Table 1. As can be seen, knowledge availability and the capability to make preventive use of problem solutions, have the highest effects on the capability to holistically detect and remedy problems across the product development process, out of the tested variables.

**Table 1:** Spearman coefficients for each of Q3, Q4, Q8 and Q9 respectively, with Q10.

*X*	*Y*	$r_{\mathrm{XY}}^{\mathrm{Sp}}$
Only the knowledge of individuals or small groups is used for the solution of complex problems. (Q5)	Capability to holistically detect and remedy problems across the product development process (Q10)	−0.05
The intended documentation methods do not (or under considerable effort) allow synergies across zones, departments and facility locations. (Q4)		−0.15
Availability of problem relevant knowledge during the product development process. (Q8)		0.39
Capability to use generated problem solutions preventively or anticipatory in the future. (Q9)		0.523
